# Supplementary material for: Shortcut barcoding and early pooling for scalable multiplex single-cell reduced-representation CpG methylation sequencing at single nucleotide resolution
Source: Nucleic Acids Res. 2023 Oct 23;51(21):e108. doi: 10.1093/nar/gkad892 (PMC10681715; doi:10.1093/nar/gkad892)
Supplement: gkad892_Supplemental_Files [file gkad892_supplemental_files.zip › Supplementary Figure.pdf]

a

Time	Hands-On Time: Bench		Hands-Off Time: Instrument		Stop & Storage
	Single cell preparation	variable			
	**Encapsulation**				
	Reagent Prep	5 min			
4.5 h			Lysis & Enzyme inactivation	45 min	
	DNA extraction	50 min			
			MspI digestion	150 min	
	Reagent Prep	10 min			
	**Pooling**				
			Adapter ligation	85 min	
	Sample pooling	30 min			
			Adapter filling<br>Conversion	30 min<br>40 min	
	Purification	30 min			
8.5 h			First-round PCR	60 min	4 ℃ < 24 h
	Purification	20 min			
			DNA digestion with BciVI<br>Adapter ligation	140 min<br>15 min	
	Purification<br>Size selection	20 min<br>40 min			
			DNA quantification (Qubit)	5 min	
	**Library output**				
			Second-round PCR	40 min	-20 ℃ > 24 h
1.5 h	Library Purification	40 min			
			DNA quantification (Qubit)	5 min	
14.5 h	**Sum**	**245 min**		**615 min**	

b

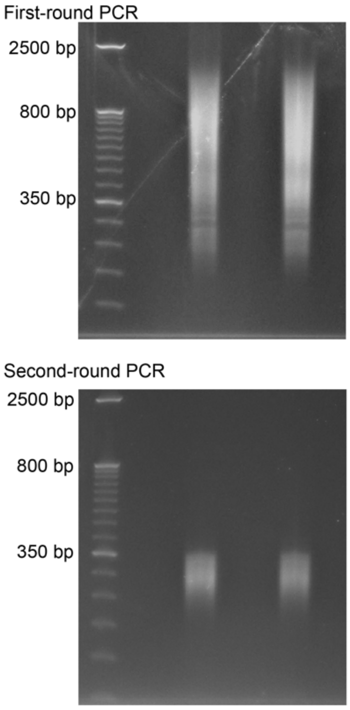

c

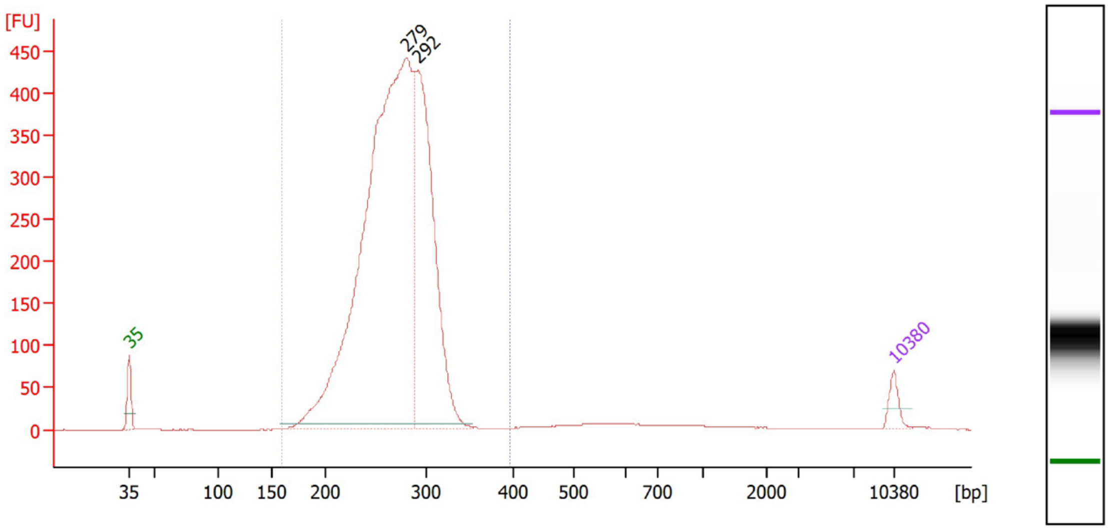

Figure S1. Critical technological designs with procedure time frame of msRRBS. (a) The standard duration of each step of msRRBS procedure. (b) E-Gel™ EX 2% agarose gel electropherograms of the first and second rounds of PCR amplification, where the library fragments recovered are 175-350 bp. (c) Bioanalyzer electropherograms of msRRBS libraries. Here the library fragments are >175 bp with a mean length near 300 bp.

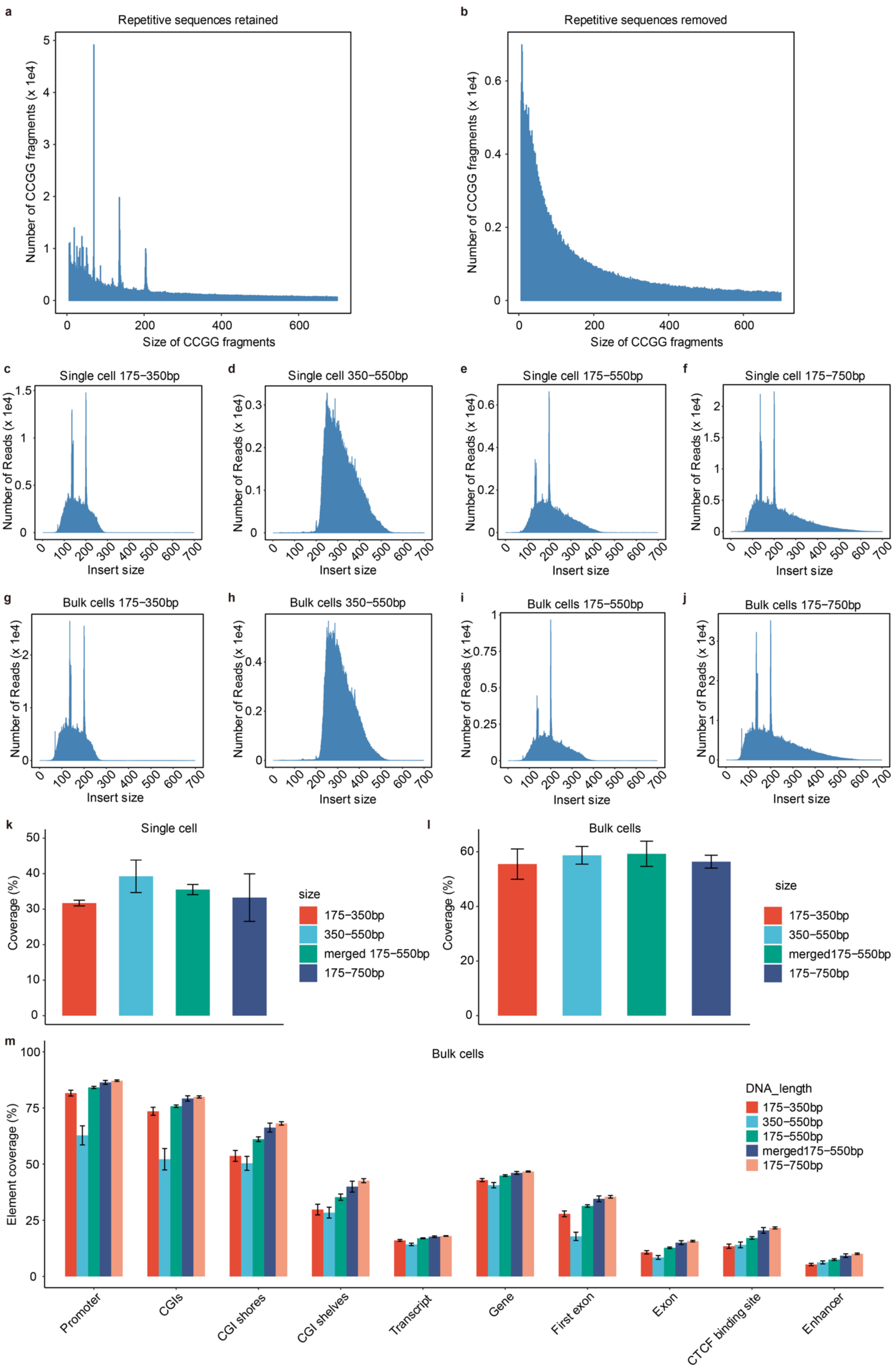

a
Repetitive sequences retained
Number of CCGG fragments (x 1e4)
Size of CCGG fragments
b
Repetitive sequences removed
Number of CCGG fragments (x 1e4)
Size of CCGG fragments
c
Single cell 175−350bp
d
Single cell 350−550bp
e
Single cell 175−550bp
f
Single cell 175−750bp
g
Bulk cells 175−350bp
h
Bulk cells 350−550bp
i
Bulk cells 175−550bp
j
Bulk cells 175−750bp
Number of Reads (x 1e4)
Insert size
k
Single cell
Coverage (%)
size
175−350bp
350−550bp
merged 175−550bp
175−750bp
l
Bulk cells
Coverage (%)
size
175−350bp
350−550bp
merged175−550bp
175−750bp
m
Bulk cells
Element coverage (%)
DNA_length
175−350bp
350−550bp
175−550bp
merged175−550bp
175−750bp
Promoter
CGIs
CGI shores
CGI shelves
Transcript
Gene
First exon
Exon
CTCF binding site
Enhancer

Figure S2. Mapping rates and coverage of elements of msRRBS/mRRBS with different ranges of library size assayed with cell line K562. (a-b) The distribution of silicon fragments of ‘CCGG’ restriction sites (ex. MspI site) with (a) and without (b) repetitive sequences in the human genome. (c-j) The distribution of the insert sizes across the various library size ranges tested of single cells (c-f) and bulk cells (g-j), with repetitive sequences removed, decoded from the sequencing data. (k-l) The average coverage of the CpG sites identified across the various library size ranges tested of single cells (k) and bulk cells (l). (m) Coverage of various genomic elements along the genome associated with different library sizes, detected in K562 bulk cells. The libraries of different sizes for bulk cells (m) and the library of 175-750bp for single-cells (k) are the results with 4 biological replicates, while the libraries of other sizes for single-cells are from 3 replicates (k).

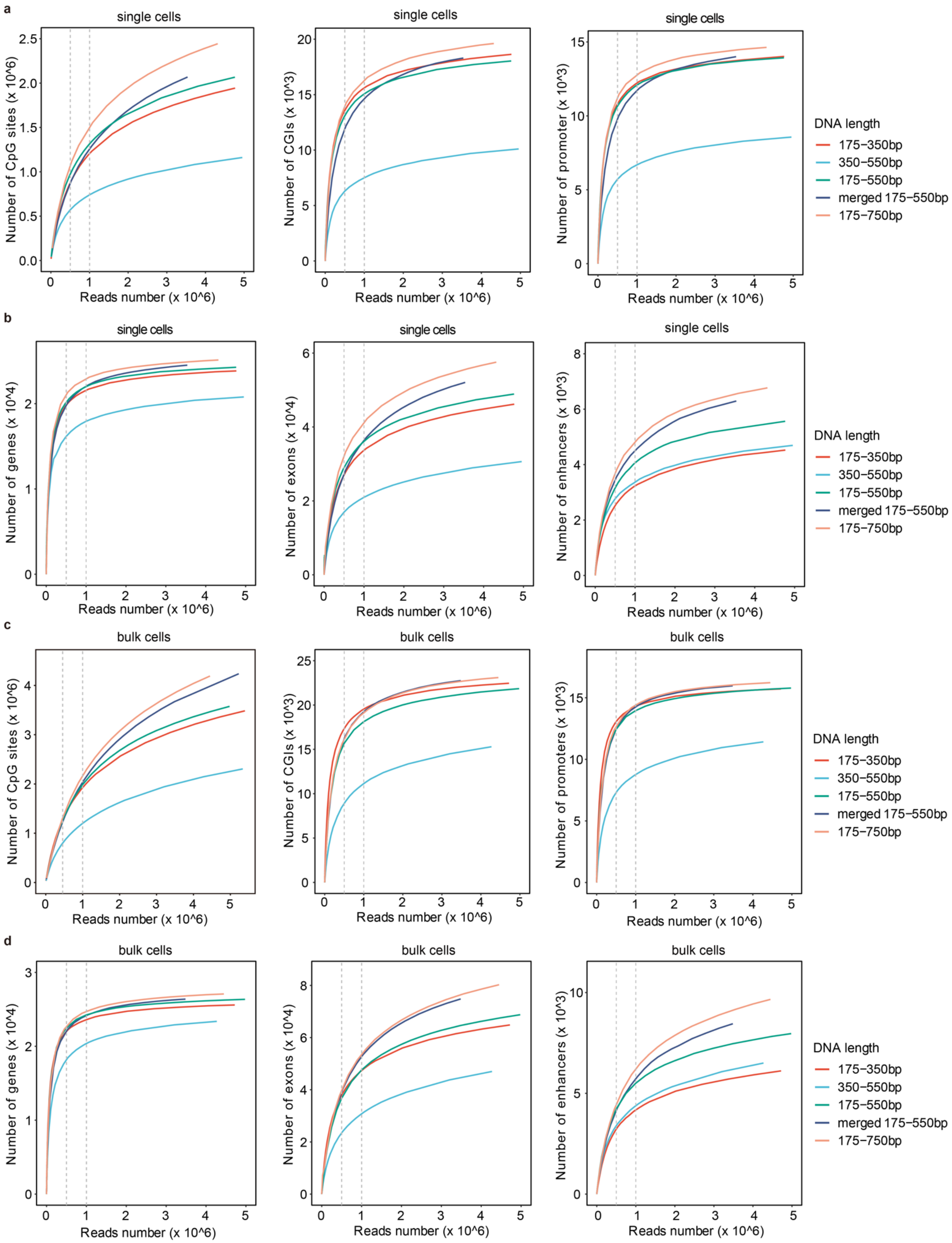

a
single cells
Number of CpG sites (x 10^6)
Reads number (x 10^6)
single cells
Number of CGIs (x 10^3)
Reads number (x 10^6)
single cells
Number of promoter (x 10^3)
Reads number (x 10^6)
DNA length
175−350bp
350−550bp
175−550bp
merged 175−550bp
175−750bp
b
single cells
Number of genes (x 10^4)
Reads number (x 10^6)
single cells
Number of exons (x 10^4)
Reads number (x 10^6)
single cells
Number of enhancers (x 10^3)
Reads number (x 10^6)
DNA length
175−350bp
350−550bp
175−550bp
merged 175−550bp
175−750bp
c
bulk cells
Number of CpG sites (x 10^6)
Reads number (x 10^6)
bulk cells
Number of CGIs (x 10^3)
Reads number (x 10^6)
bulk cells
Number of promoters (x 10^3)
Reads number (x 10^6)
DNA length
175−350bp
350−550bp
175−550bp
merged 175−550bp
175−750bp
d
bulk cells
Number of genes (x 10^4)
Reads number (x 10^6)
bulk cells
Number of exons (x 10^4)
Reads number (x 10^6)
bulk cells
Number of enhancers (x 10^3)
Reads number (x 10^6)
DNA length
175−350bp
350−550bp
175−550bp
merged 175−550bp
175−750bp

Figure S3. Saturation analysis for variant genomic elements in different sizes of recovered fragments of msRRBS/mRRBS libraries with cell line K562. (a-d) Number of CpG sites, CpG islands, promoters, genes, exons, and enhancers with different sequencing depth detected in K562 across the various library size ranges analyzed in single cells (a-b) and bulk cells (c-d). The vertical gray dashed lines denote 0.5 M and 1 M clean reads as reference lines for comparison.

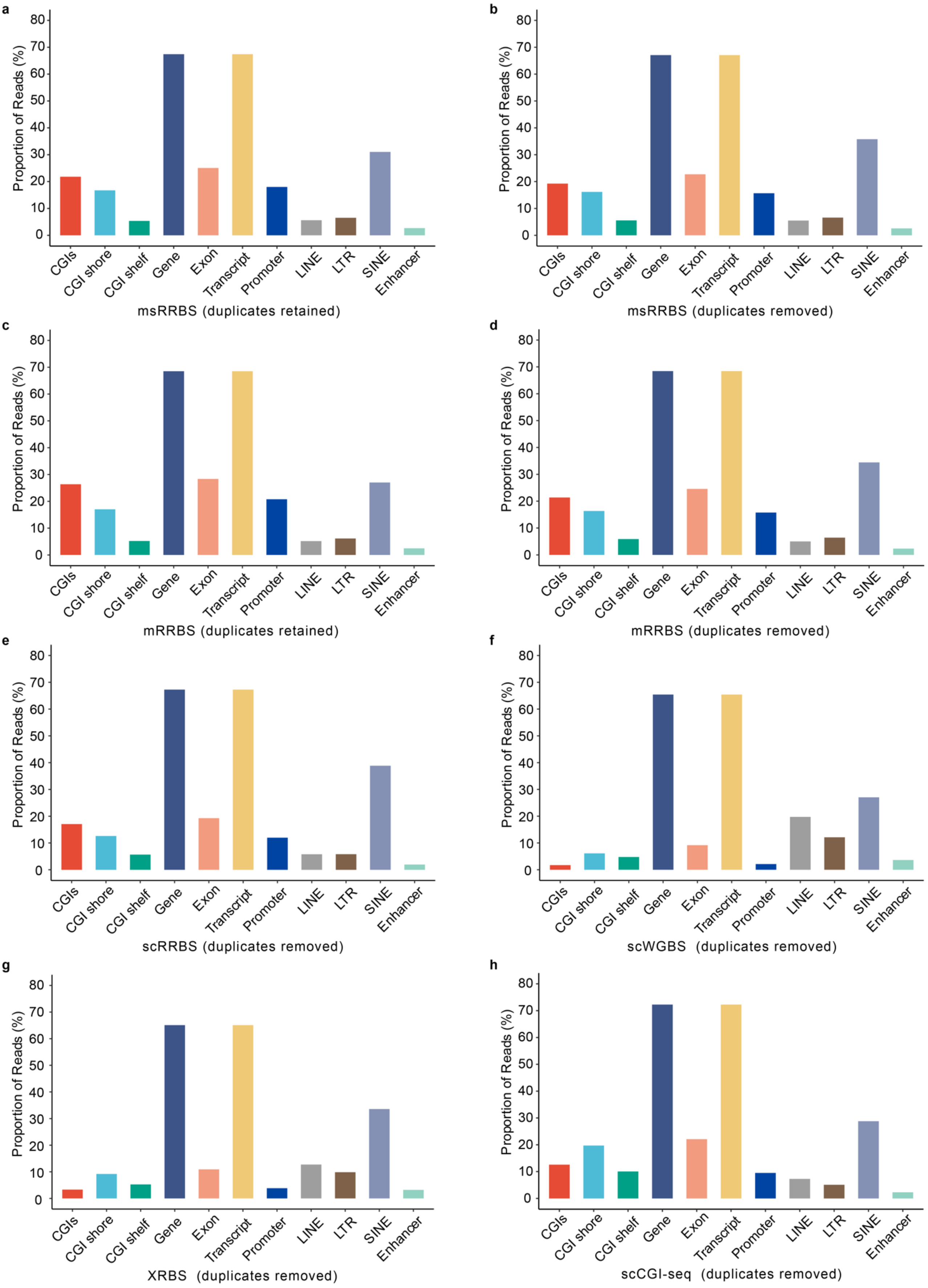
a
Proportion of Reads (%)
0
10
20
30
40
50
60
70
80
CGIs
CGI shore
CGI shelf
Gene
Exon
Transcript
Promoter
LINE
LTR
SINE
Enhancer
msRRBS (duplicates retained)
b
Proportion of Reads (%)
msRRBS (duplicates removed)
c
Proportion of Reads (%)
mRRBS (duplicates retained)
d
Proportion of Reads (%)
mRRBS (duplicates removed)
e
Proportion of Reads (%)
scRRBS (duplicates removed)
f
Proportion of Reads (%)
scWGBS (duplicates removed)
g
Proportion of Reads (%)
XRBS (duplicates removed)
h
Proportion of Reads (%)
scCGI-seq (duplicates removed)

Figure S4. High efficiency of msRRBS in targeting CpG islands and promoters. (a-h) The proportion of reads associated with promoters, CpG islands, gene bodies and other genomic elements in msRRBS with library sizes of 175-350 bp (a-d), conventional scRRBS (e), WGBS (f), XRBS (g) and scCGI-seq (h) **(Supplementary table 5)**. A single cell data is randomly picked up from the corresponding database for this analysis. It is noted that there are intersections between various genomic elements in the genome, particularly CGIs locate in genes, promoters and other genomic elements, so the total proportion of reads falling between various genomic elements is more than 100% **(Supplementary Figure 5a)**.

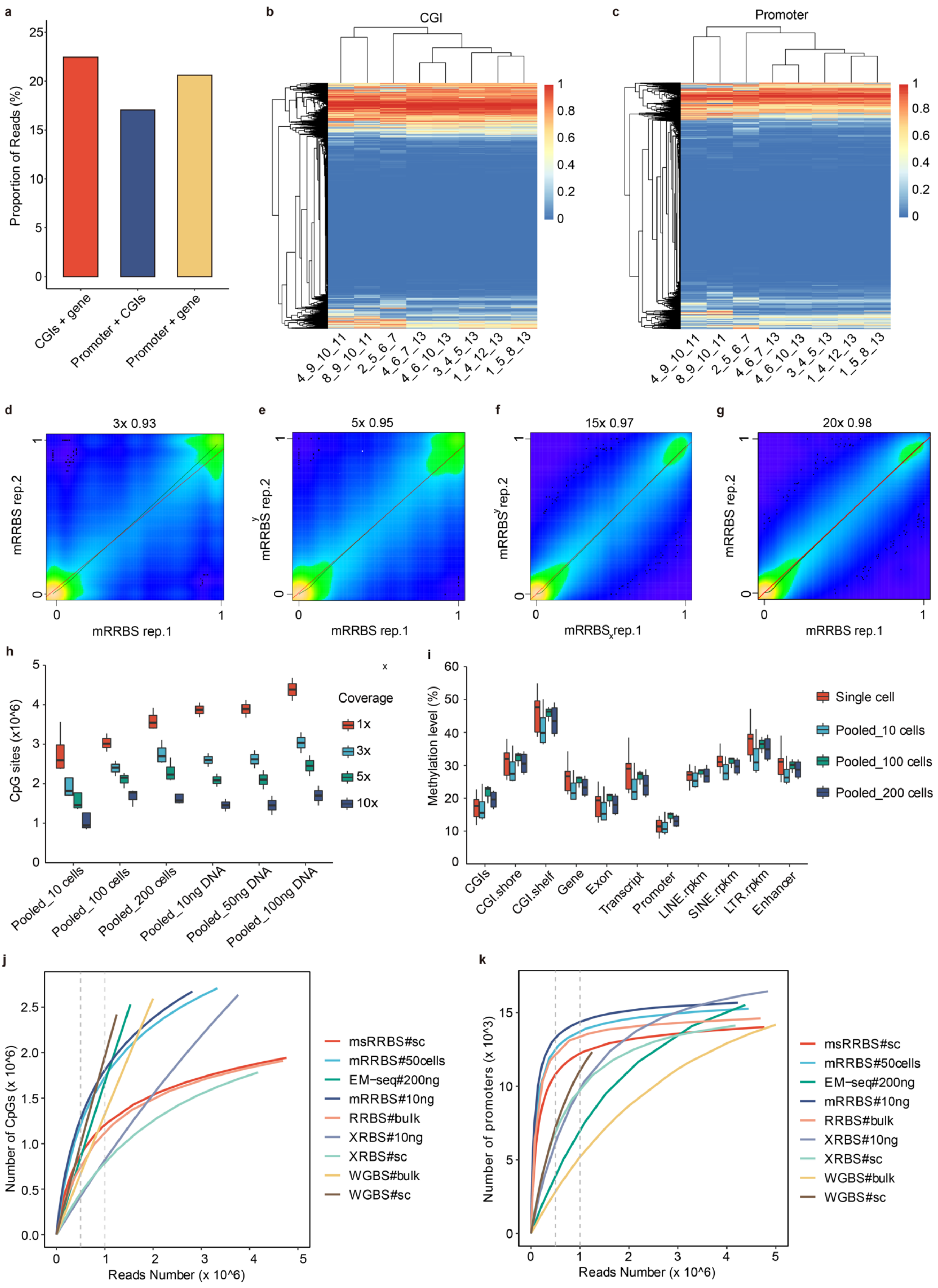
a
Proportion of Reads (%)
0
5
10
15
20
25
CGIs + gene
Promoter + CGIs
Promoter + gene
b
CGI
c
Promoter
1
0.8
0.6
0.4
0.2
0
4_9_10_11
8_9_10_11
2_5_6_7
4_6_7_13
4_6_10_13
3_4_5_13
1_4_12_13
1_5_8_13
d
3x 0.93
e
5x 0.95
f
15x 0.97
g
20x 0.98
mRRBS rep.2
mRRBS rep.1
h
CpG sites (x10^6)
Coverage
1x
3x
5x
10x
Pooled_10 cells
Pooled_100 cells
Pooled_200 cells
Pooled_10ng DNA
Pooled_50ng DNA
Pooled_100ng DNA
i
Methylation level (%)
Single cell
Pooled_10 cells
Pooled_100 cells
Pooled_200 cells
CGIs
CGI.shore
CGI.shelf
Gene
Exon
Transcript
Promoter
LINE.rpkm
SINE.rpkm
LTR.rpkm
Enhancer
j
Number of CpGs (x 10^6)
Reads Number (x 10^6)
msRRBS#sc
mRRBS#50cells
EM-seq#200ng
mRRBS#10ng
RRBS#bulk
XRBS#10ng
XRBS#sc
WGBS#bulk
WGBS#sc
k
Number of promoters (x 10^3)
Reads Number (x 10^6)

Figure S5. Coverage of genomic elements of msRRBS assayed with cell line K562. (a) The proportion of overlapping reads among CGIs, promoters and genes; '+' represents overlapping between the 2 elements. (b-c) Heatmap shows the methylation levels of CGIs (b) and promoters (c) in four randomly in silico-merged single cells from cell line K562. The number on the x axis represents the barcode of the merged single cells. (d-g) Pearson correlation heatmap compares the methylation pattern based on CpG sites, acquired by mRRBS, between technical replicates of bulk cells for cell line K562 in coverage ⩾ 3× (d, R = 0.93), ⩾ 5× (e, R = 0.95), ⩾ 15× (f, R = 0.97) and ⩾ 20× (g, R = 0.98). (h) Number of CpG sites detected in micro-bulk (10-200 cells, n=4) and extracted gDNA (10-100ng, n=2) of K562 with different depths of coverage. (i) Methylation levels in different genomic elements of single cells (n=15), micro-bulk cells of 10, 100, and 200 cells (n=2) of K562. (j-k) Number of CpG sites (j) and promoters (k) containing CpG islands with at least 1 CpG site covered as a function of sequencing depth for msRRBS, mRRBS, conventional RRBS, WGBS, XRBS and EM-Seq in cell line K562. The vertical gray dashed lines denote 0.5 M and 1 M clean reads as reference lines for comparison.

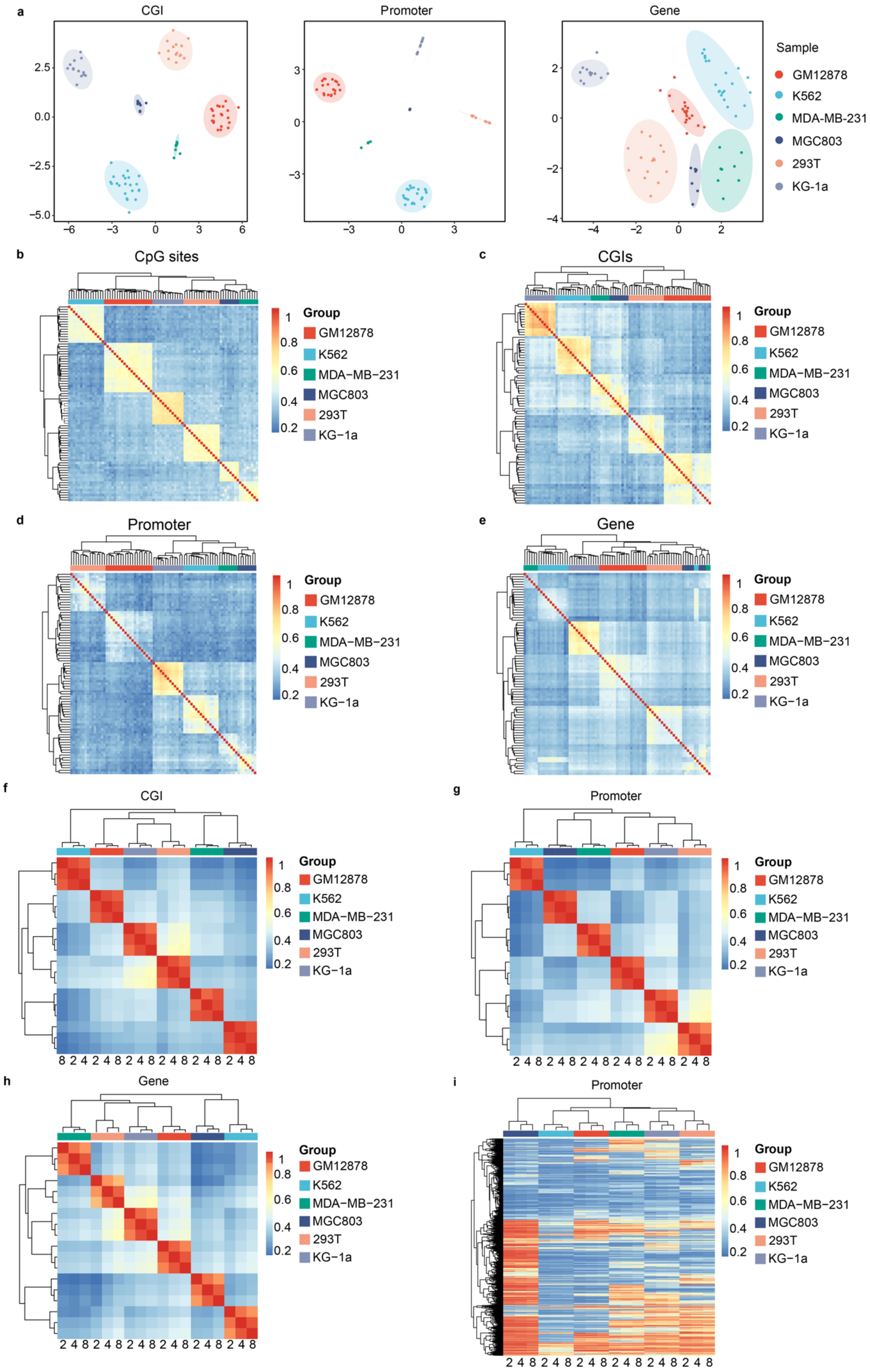

a
CGI
Promoter
Gene
Sample
GM12878
K562
MDA-MB-231
MGC803
293T
KG-1a
b
CpG sites
c
CGIs
d
Promoter
e
Gene
f
CGI
g
Promoter
h
Gene
i
Promoter
Group
GM12878
K562
MDA−MB−231
MGC803
293T
KG−1a

Figure S6. Distinct methylation profiling of six cell lines in variant genomic elements examined by msRRBS. (a) Unsupervised clustering of the single cells from the six cell lines based on methylation levels of CGIs, promoters, and genes, generated with tSNE. (b-e) Pearson correlation heatmap shows the methylation levels of CpG sites(b), CGIs (c), promoters (d), and genes(e) among the single-cell samples. The color key from blue to red indicates low to high correlation. Six cell lines: 293T (n=15), GM12878 (n=20), K562 (n=15), KG-1a (n=13), MDA-MB-231 (n=8) and MGC803 (n=8) were analyzed. (f-h) Pearson correlation heatmap shows the methylation levels of CGIs (f), promoters (g), and genes (h) of 2, 4, and 8 single cells in the corresponding elements were randomly in silico-merged from each of the six cell lines. (i) Methylation profiles of 2, 4, and 8 single cells in the promoter were randomly in silico-merged for each of the six cell lines. The number 0 represents unmethylated, and the number 1 represents fully methylated. The color changes from blue to red to indicate a gradual increase in methylation level.

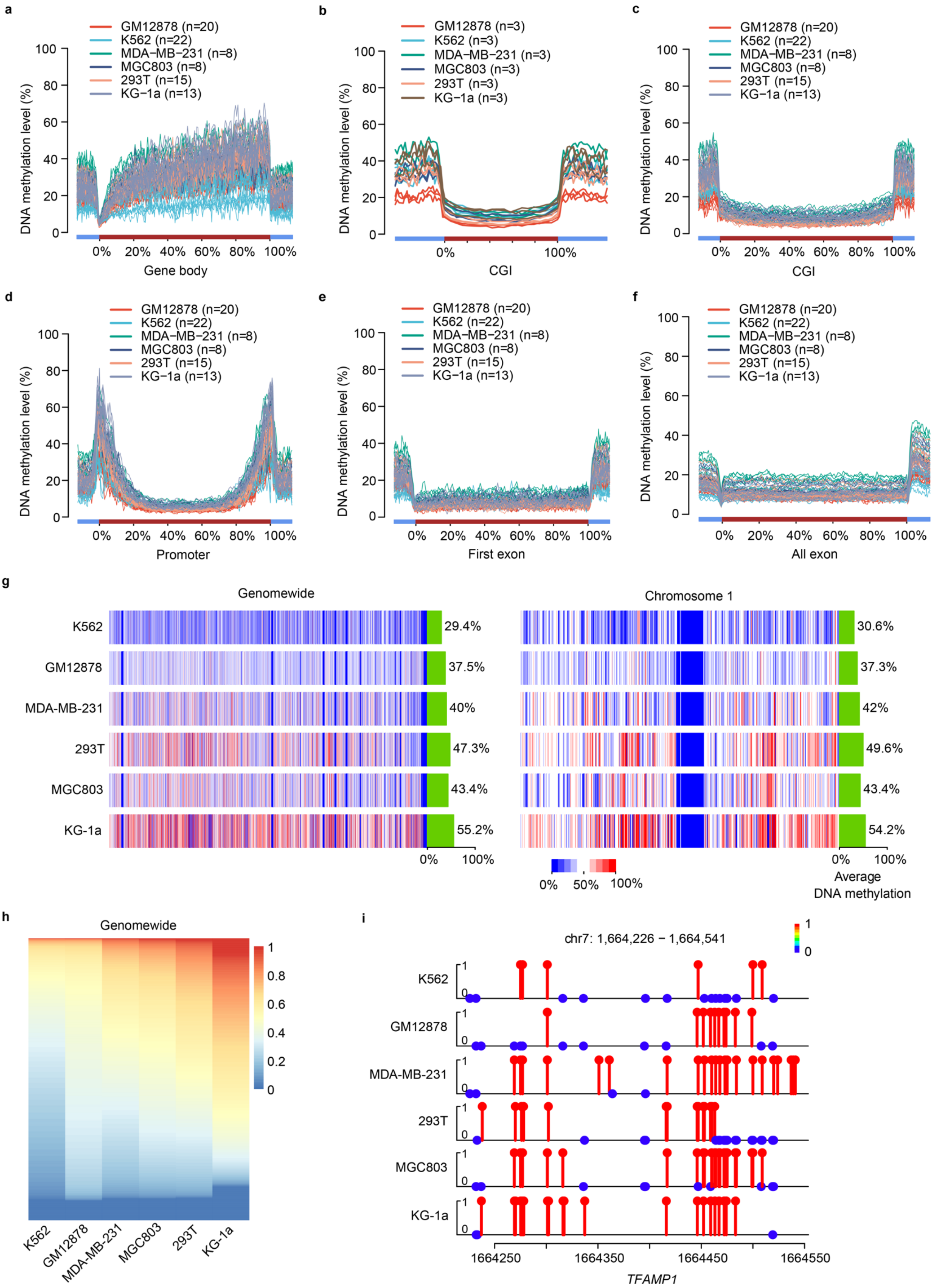

a
GM12878 (n=20)
K562 (n=22)
MDA−MB−231 (n=8)
MGC803 (n=8)
293T (n=15)
KG−1a (n=13)
DNA methylation level (%)
Gene body
b
GM12878 (n=3)
K562 (n=3)
MDA−MB−231 (n=3)
MGC803 (n=3)
293T (n=3)
KG−1a (n=3)
CGI
c
CGI
d
Promoter
e
First exon
f
All exon
g
Genomewide
Chromosome 1
K562
GM12878
MDA-MB-231
293T
MGC803
KG-1a
29.4%
37.5%
40%
47.3%
43.4%
55.2%
30.6%
37.3%
42%
49.6%
43.4%
54.2%
0% 50% 100%
Average
DNA methylation
h
Genomewide
K562
GM12878
MDA-MB-231
MGC803
293T
KG-1a
i
chr7: 1,664,226 − 1,664,541
1664250
1664350
1664450
1664550
TFAMP1

Figure S7. Reliability and reproducibility of msRRBS in the measurement of methylation pattern. (a-f) DNAme patterns across gene bodies (a), CGIs (b and c), promoters (d), first exons (e), all exons (f) and 5 kb upstream and downstream of the TSSs and the TESs of all RefSeq genes in combination. (g-h) DNAme levels of whole genome (g and h, the methylation levels of figure h are sorted) and chromosome 1 (g, right) for each cell line with 8 single cells in silico-merged by windowing method with at least 20 CpG sites per 50-kb bin. (i) Methylation status of six cell lines in a representative window with gene TFAMP1 (an example arbitrarily selected). The methylation levels of most of the CpG sites of KG-1a in the window are fully methylated (Red lollipop), and most of the CpG sites of K562 in the window are unmethylated (blue circles).

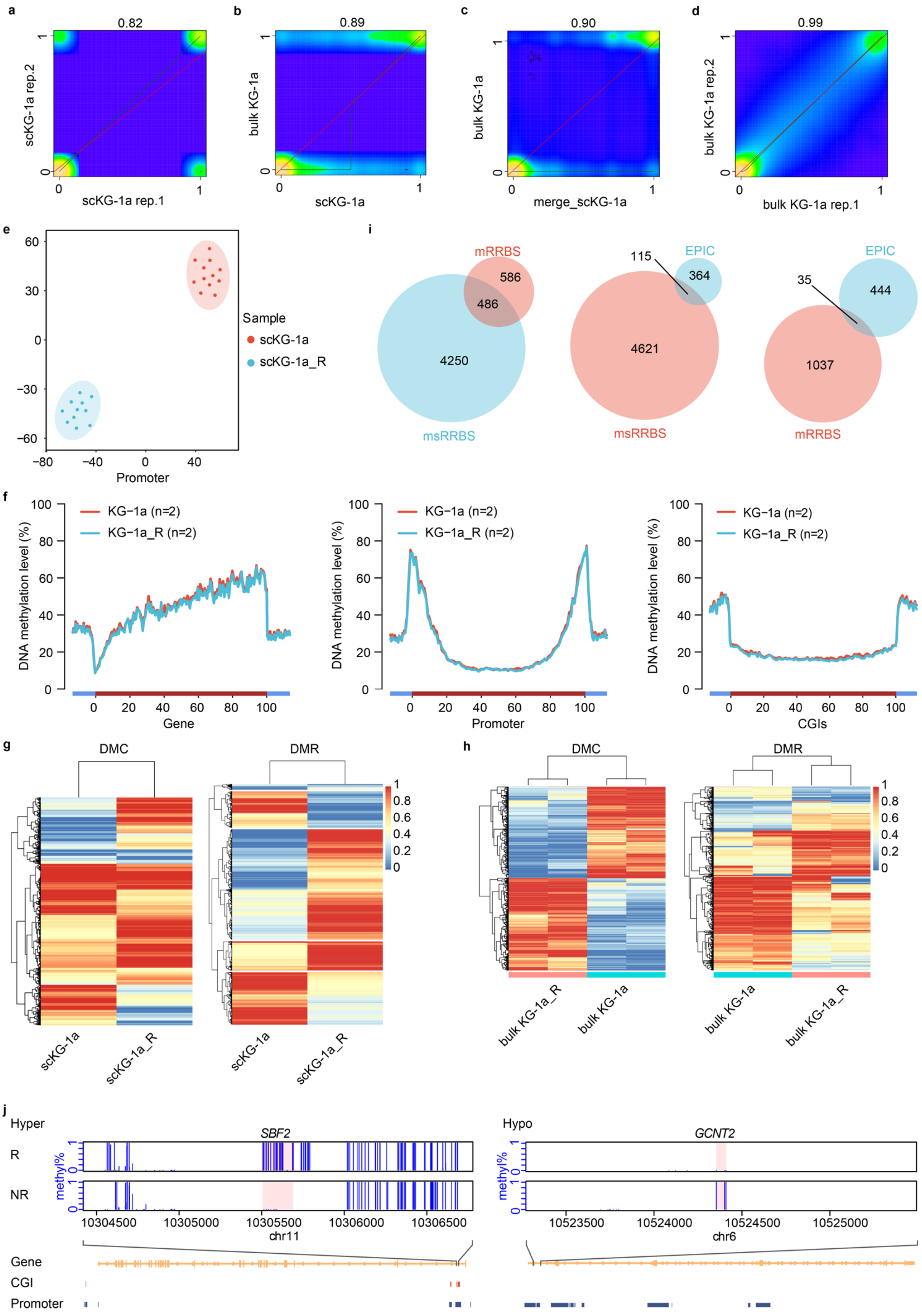

a
0.82
scKG-1a rep.2
scKG-1a rep.1
b
0.89
bulk KG-1a
scKG-1a
c
0.90
bulk KG-1a
merge_scKG-1a
d
0.99
bulk KG-1a rep.2
bulk KG-1a rep.1
e
Promoter
Sample
scKG-1a
scKG-1a_R
i
mRRBS
586
486
4250
msRRBS
115
EPIC
364
4621
msRRBS
35
EPIC
444
1037
mRRBS
f
DNA methylation level (%)
KG−1a (n=2)
KG−1a_R (n=2)
Gene
Promoter
CGIs
g
DMC
DMR
scKG-1a
scKG-1a_R
h
DMC
DMR
bulk KG-1a_R
bulk KG-1a
j
Hyper
SBF2
R
NR
methyl%
chr11
Hypo
GCNT2
chr6
Gene
CGI
Promoter

Figure S8. msRRBS study on KG-1a resistant to cytarabine (KG-1a_R) versus the original KG-1a. (a-d) Pearson correlation heatmap compares methylation values of individual CpG sites acquired by msRRBS between two single cells (a, R = 0.82), between a single cell and a bulk of cells (b, R = 0.89), between pseudo-bulk (merged 13 cells) and a bulk of cells (c, 0.90), and between technical replicates of bulk cells (d, R = 0.99) for the original culture of cell line KG-1a. (e) Unsupervised clustering of single cells based on the methylation of promoters for KG-1a_R (n=11) and KG-1a (n=12), generated by tSNE. (f) Average DNAme patterns across gene bodies, promoters, and CGIs from the data set of bulk cells by mRRBS. (g-j) KG-1a_R versus KG-1a cells. (g) DMC methylation heatmap for in silico-merged single cells of KG-1a. (h) DMC and DMR methylation heatmap for bulk cells from KG-1a_R versus KG-1a. (i) Hypo-DMR intersections of KG-1a_R vs. KG-1a among methods msRRBS, mRRBS and EPIC methylation array. (j) Two windows each contain a DMR of merged single cells from KG-1a_R versus the corresponding KG-1a population (MWU-test p-value < 0.05, number of CpG sites in one DMR ≥ 3, the distance between two independent DMRs $\geq$ 100bp, length of DMR $\geq$ 50bp).

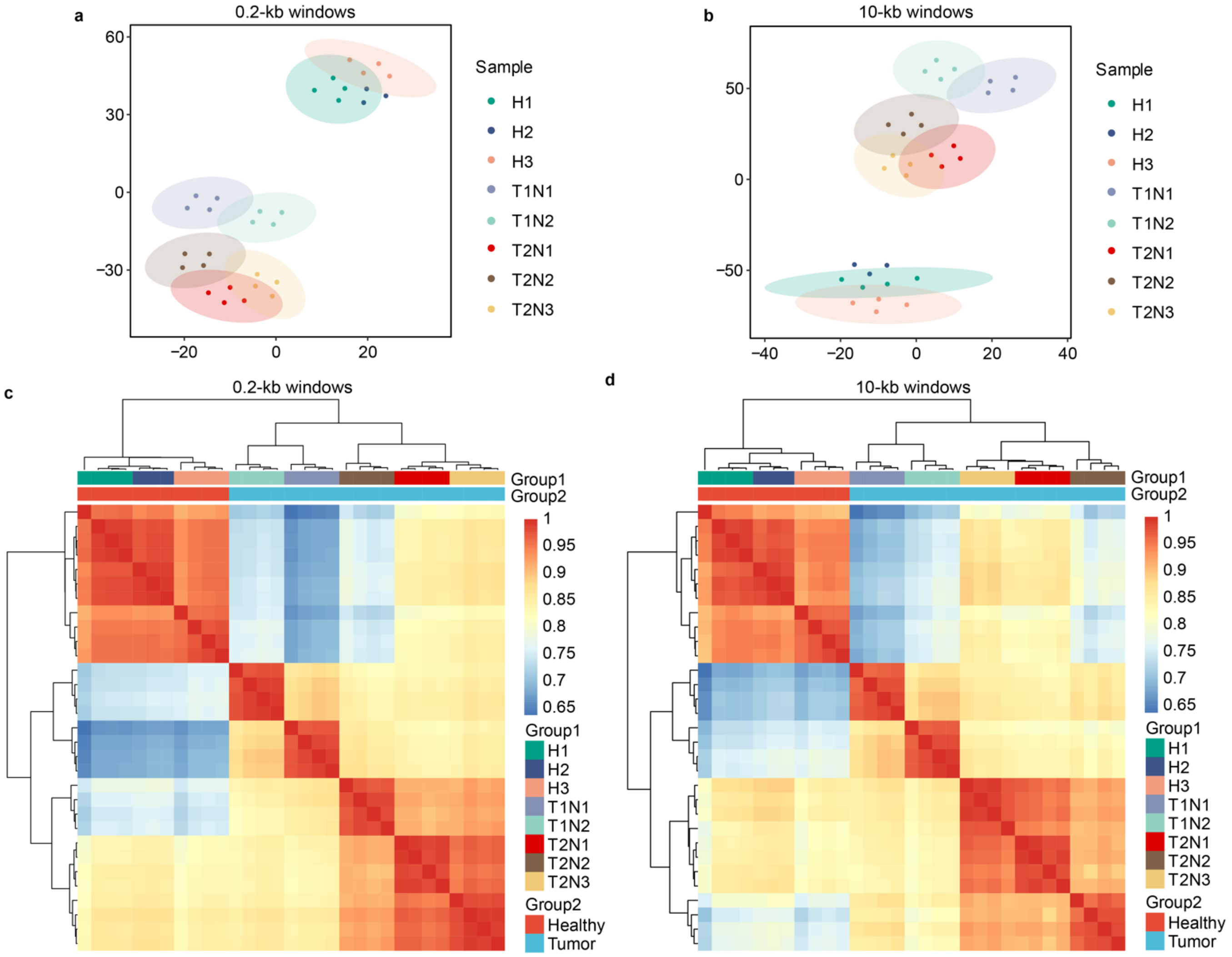

a
0.2-kb windows
Sample
H1
H2
H3
T1N1
T1N2
T2N1
T2N2
T2N3
b
10-kb windows
c
0.2-kb windows
Group1
Group2
Healthy
Tumor
d
10-kb windows

Figure S9. Clustering and correlation of hepatocellular carcinoma (HCC) samples in mice uncovered by msRRBS based on 2 additional size windows. (a-d) Analysis of HCC bulk cells and their controls based on methylation levels in 0.2-kb (a and c) and 10-kb (b and d) windows over the genome for two HCC mice with two or three liver cancer nodules versus three liver biopsies from healthy mice. Except for H2 with 3 technical replicates, all the other samples were measured with 4 technical replicates. (a-b) Unsupervised clustering with tSNE. (c-d) Pearson correlation heatmap. The color key from blue to red indicates low to high correlation.

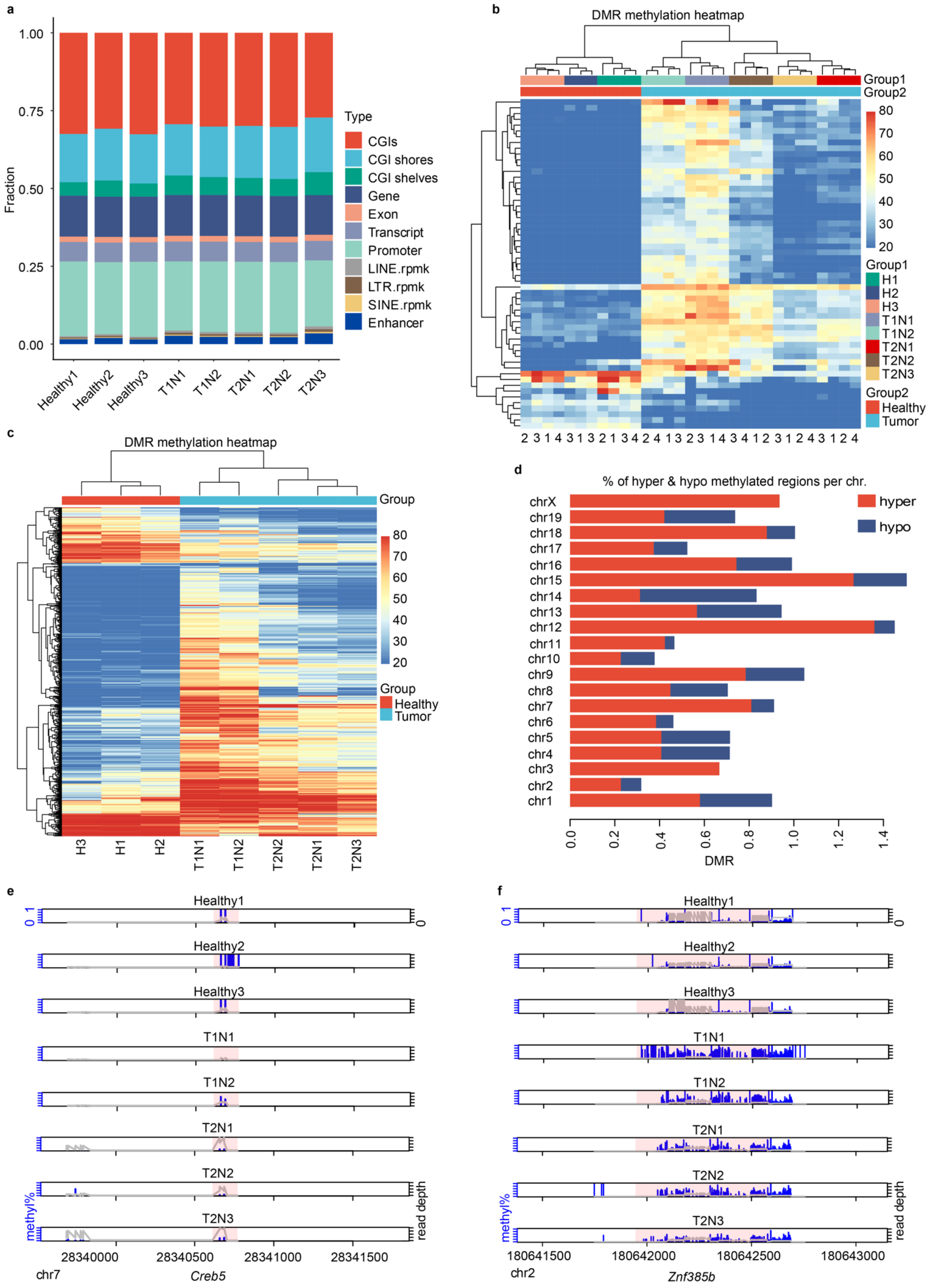

a
Fraction
Type
CGIs
CGI shores
CGI shelves
Gene
Exon
Transcript
Promoter
LINE.rpmk
LTR.rpmk
SINE.rpmk
Enhancer
Healthy1
Healthy2
Healthy3
T1N1
T1N2
T2N1
T2N2
T2N3
b
DMR methylation heatmap
Group1
Group2
H1
H2
H3
T1N1
T1N2
T2N1
T2N2
T2N3
Healthy
Tumor
c
DMR methylation heatmap
Group
Healthy
Tumor
H3
H1
H2
T1N1
T1N2
T2N2
T2N1
T2N3
d
% of hyper & hypo methylated regions per chr.
hyper
hypo
DMR
e
Healthy1
Healthy2
Healthy3
T1N1
T1N2
T2N1
T2N2
T2N3
methyl%
read depth
28340000
28340500
28341000
28341500
chr7
Creb5
f
Healthy1
Healthy2
Healthy3
T1N1
T1N2
T2N1
T2N2
T2N3
methyl%
read depth
180641500
180642000
180642500
180643000
chr2
Znf385b

Figure S10. Additional features and DMR examples of mRRBS analysis for the hepatocellular carcinoma (HCC) in mice. (a) Average detection rates of different genomic elements in each sample. (b-c) DMR methylation heatmap for individual samples (b) or in silico-merge of technical replicates (c). Each sample was measured with 3 or 4 technical replicates. (e-f) Two representative DMRs between HCC and liver from healthy mice (MWU-test p-value < 0.05, number of CpG sites in one DMR ≥ 3, the distance between two independent DMRs $\geq$ 100bp, length of DMR $\geq$ 50bp). (d) Percentage of hyper & hypo methylated regions per chromosome, q value<0.01 & methylation diff. >=25 %. (e) *Creb5* is hypermethylated in healthy mice and hypomethylated in HCC mice. (f) *Znf385b* is hypomethylated in healthy mice and hypermethylated in HCC mice. The number 0 represents unmethylated, and the number 1 represents methylated. The pink background represents the length of the DMR region. The blue vertical bars represent methylation levels, and higher vertical bars represent higher methylation levels. The higher the gray line is, the deeper the reads are.
